# Roles of thermostable direct hemolysin (TDH) and TDH-related hemolysin (TRH) in *Vibrio parahaemolyticus*

**DOI:** 10.3389/fmicb.2014.00805

**Published:** 2015-01-22

**Authors:** Pendru Raghunath

**Affiliations:** Department of Microbiology, Dr. VRK Women’s Medical College Teaching Hospital and Research CentreHyderabad, India

**Keywords:** *V. parahaemolyticus*, *tdh*, *trh*, virulence factors

## Abstract

*Vibrio parahaemolyticus* is the leading cause of seafood borne bacterial gastroenteritis in the world, often associated with the consumption of raw or undercooked seafood. However, not all strains of *V. parahaemolyticus* are pathogenic. The thermostable direct hemolysin (TDH) or TDH-related hemolysin (TRH) encoded by *tdh* and *trh* genes, respectively, are considered major virulence factors in *V. parahaemolyticus*. However, about 10% of clinical strains do not contain *tdh* and/or *trh*. Environmental isolates of *V. parahaemolyticus* lacking *tdh* and/or *trh* are also highly cytotoxic to human gastrointestinal cells. Even in the absence of these hemolysins, *V. parahaemolyticus* remains pathogenic indicating other virulence factors exist. This mini review aims at discussing the possible roles of *tdh* and *trh* genes in clinical and environmental isolates of *V. parahaemolyticus*.

## INTRODUCTION

*Vibrio parahaemolyticus* is a Gram-negative, halophilic bacterium that occurs in estuarine environments worldwide (Su and Liu, 2007; Nelapati et al., 2012; Ceccarelli et al., 2013; Zhang and Orth, 2013). It is the leading cause of seafood borne bacterial gastroenteritis in the world, often associated with the consumption of raw or undercooked seafood. However, not all strains of *V. parahaemolyticus* are pathogenic. Although mechanism by which *V. parahaemolyticus* causes enteric disease is not fully understood, clinical isolates most often produce either the thermostable direct hemolysin (TDH) or TDH-related hemolysin (TRH) encoded by *tdh* and *trh* genes, respectively (Zhang and Austin, 2005). TDH and TRH are considered major virulence factors in *V. parahaemolyticus* (Ceccarelli et al., 2013).

## CORRELATION OF *tdh* AND *trh* WITH THE VIRULENCE OF *V. parahaemolyticus*

Thermostable direct hemolysin exerts a variety of biological activities such as hemolytic activity, cytotoxicity, cardiotoxicity, and enterotoxicity. TDH is a pore-forming toxin, forms pores of ~2 nm in diameter on erythrocyte membrane (Matsuda et al., 2010). The fairly large pore size allows both water and ions to flow through the membrane (Honda et al., 1992). These alterations in ion flux in the intestine is responsible for the diarrhea observed during infection. TRH is a heat labile toxin and immunologically similar to TDH (Honda et al., 1988). Both genes, *trh* and *tdh* share approximately 70% homology (Kishishita et al., 1992). Similar to TDH, TRH also activates cl^-^ channels resulting in altered ion flux (Takahashi et al., 2000). Although TDH and TRH correlate with pathogenic strains, they do not fully account for *V. parahaemolyticus* pathogenicity (Lynch et al., 2005). Several studies have reported that some of the clinical strains do not contain *tdh* and/or *trh* (Jones et al., 2012; Li et al., 2014; Pazhani et al., 2014). Even in the absence of these hemolysins, *V. parahaemolyticus* remains pathogenic indicating other virulence factors exist (Jones et al., 2012). Mahoney et al. (2010) reported that environmental isolates of *V. parahaemolyticus* lacking *tdh* and/or *trh* produced putative virulence factors like extracellular proteases, biofilm, siderophore, and highly cytotoxic to human gastrointestinal cells. Park et al. (2004a) reported that deletion of both copies of *tdh* did not affect the cytotoxicity to HeLa cells and enterotoxicity assayed by the rabbit ileal loop test was lowered by *tdh* deletion, but the mutant still showed partial fluid accumulation in rabbit intestine. Ming et al. (1994) reported that *trh* deletion resulted in partial but apparent fluid accumulation in ligated rabbit small intestine. These results clearly indicate that cytotoxicity and enterotoxicity of pathogenic *V. parahaemolyticus* are not explained by TDH and TRH alone and suggest that an unkown virulence factor (s) could be responsible for pathogenicity.

## TYPE III SECRETION SYSTEMS

The type III secretion system (T3SS) of *V. parahaemolyticus* has been suggested as an important virulence factor is (Shimohata and Takahashi, 2010; Broberg et al., 2011). Two non-redundant T3SSs are reported from many *V. parahaemolyticus* strains (Park et al., 2004b; Broberg et al., 2011). Many studies suggest that T3SS1 is responsible for cytotoxicity, mouse lethality, and possibly induction of autophagy (Park et al., 2004b; Burdette et al., 2009; Hiyoshi et al., 2010). T3SS2 appears to be responsible for enterotoxicity and may play a role in the environmental fitness of strains (Park et al., 2004b; Hiyoshi et al., 2010; Matz et al., 2011). All *V. parahaemolyticus* isolates possess T3SS1 (Park et al., 2004b; Noriea et al., 2010). While T3SS2 is commonly associated with *V. parahaemolyticus* carrying *tdh* and/or *trh*. Two distinct lineages of T3SS2 have been described, showing correlations of *tdh* with T3SS2α and *trh* with T3SS2β (Park et al., 2004b; Noriea et al., 2010). However, recently T3SS2β has been detected in *tdh*- and *trh*-negative environmental strains of *V. parahaemolyticus* (Paranjpye et al., 2012). In another study, authors screened 77 clinical isolates of *V. parahaemolyticus*, which were submitted to the Centers for Disease Control and Prevention (CDC) in 2007 from wound infections or food-borne illness and reported that 21 of 77 (27%) clinical *V. parahaemolyticus* strains were negative for *tdh*, *trh*, and T3SS2 (Jones et al., 2012). The results of these studies raise some concerns about the reliability of the *tdh*, *trh*, and T3SS2 genes as predictors of overall strain virulence.

## TYPE VI SECRETION SYSTEMS

Comparison between pandemic and non-pandemic strains of *V. parahaemolyticus* led to identification of type VI secretion systems, T6SS1 (VP1386–VP1420) and T6SS2 (VPA1030–VPA1043), located on chromosome 1 and 2 of *V. parahaemolyticus* RIMD 2210633, respectively, (Boyd et al., 2008; Izutsu et al., 2008). The role of T6SS2 is under investigation, preliminary data suggested that the T6SS2 is not involved in cytotoxicity, helps in adhesion to host cells (Yu et al., 2012). Since T6SS2 and T3SS2 systems co-exist, it is proposed that both systems might cooperate during an infection process in host. T6SS2 initiates the first step of infection by adhering to host cells and T3SS2 exports effector molecules by inducing enterocytotoxicity (Yu et al., 2012). Role of T6SS1 has not yet been demonstrated. Recently, researchers suggested the role of T6SSs in environmental fitness of *V. parahaemolyticus*. Salomon et al. (2013) reported that T6SS1 is most active under warm marine-like conditions, while T6SS2 is active under low salt conditions. T6SS was used as a virulence marker to differentiate *V. parahaemolyticus* strains. Chao et al. (2010) reported that most pandemic strains isolated in China had the complete set of T6SS genes, whereas the majority of non-pathogenic strains had a partial set of T6SS genes.

## POSSIBLE ROLES OF *tdh* AND *trh* IN CLINICAL AND ENVIRONMENTAL ISOLATES OF *V. parahaemolyticus*

First outbreak of foodborne gastroenteritis due to *V. parahaemolyticus* was reported in the year 1951 in Osaka, where people frequently consume raw or uncooked seafood (Fujino et al., 1953). Since then *V. parahaemolyticus* has reported from many food poisoning cases in Japan (Su and Liu, 2007; Hara-Kudo et al., 2012), in Taiwan (Yu et al., 2013), in China (Li et al., 2014), Bangladesh (Bhuiyan et al., 2002), HongKong, and Indonesia (Matsumoto et al., 2000). In India, a recent study, reported that 178 *V. parahaemolyticus* strains were isolated from 13,607 diarrheal patients admitted in Infectious Diseases Hospital, Kolkata since 2001–2012 (Pazhani et al., 2014). Kanungo et al. (2012) have reported *V. parahaemolyticus* diarrheal cases from the urban slums of Kolkata, India.

The majority of clinical cases of *V. parahaemolyticus* have been associated with *V. parahaemolyticus* strains carrying *tdh* and/or *trh* (Kanungo et al., 2012; Li et al., 2014; Pazhani et al., 2014). However, pathogenic strains including the pandemic clone have been rarely isolated from seafood and other environmental samples. This could be due to the occurrence of pathogenic strains in the estuarine environment at a lower level compared to non-pathogenic strains or that the pathogenic stains are more sensitive to dystrophic conditions in the aquatic environment and rapidly become non-culturable (Pace and Chai, 1989; Alam et al., 2002). We thought that host factors like bile or its component bile acids might trigger release from dormancy and increase virulence in *V. parahaemolyticus* strains. This could be the reason for selection of pathogenic strains in human gastrointestinal system inspite of low prevalence in aquatic environment. With this in view, a new enrichment broth containing bile salt, sodium taurocholate (ST broth) has been formulated and the efficiency of new enrichment broth in detecting and recovering pathogenic *V. parahaemolyticus* from seafood was compared with the traditional APW. Results of the study suggested that the ST broth is superior to APW for detection and isolation of pathogenic *V. parahaemolyticus* from seafood.

Previous studies suggested that expression of *tdh* gene is upregulated under conditions simulating those in the human intestine (Gotoh et al., 2010; Broberg et al., 2011). Gotoh et al. (2010) are reported that TDH and T3SS2 proteins were detected in much higher concentrations when bacteria were cultured at 37 and 42°C, which corresponds to the temperature of the intestine, than at lower temperatures. In the same study, they also identified bile as a potent stimulator of the production of TDH and T3SS2 proteins (Gotoh et al., 2010). Pathogenic strains may produce TDH and/or TRH more abundantly in hostile environment and these toxins might be helpful to acquire nutrients from host cells through their cytotoxic activities. This could be the reason for selection of pathogenic strains in human intestine compared to non-pathogenic strains. Within the human host, whether TDH and TRH play any other roles other than cytotoxicity and enterotoxicity of *V. parahaemolyticus* need to be studied. Bacterial pathogens frequently use environmental cues to discriminate between host and non-host environments. In response to these environmental cues, bacteria regulate their virulence gene expression for more efficient utilization of bacterial resources and facilitate colonization, leading to infection. *V. parahaemolyticus*, upon reaching a human host from environment, should expose to number of environmental cues such as temperature, pH, osmolarity, oxygen levels, carbon sources, and concentration of various ions and compounds. In response to these environmental cues, *V. parahaemolyticus* carrying *tdh* and/or *trh* might tightly coordinate their virulence associated genes expression. Whereas, *V. parahaemolyticus* strains lacking *tdh* and/or *trh* may not be able to regulate their virulence associated genes expression and not able to establish colonization and infection in the human host. Mahoney et al. (2010) reported that in clinical strains carrying *tdh* and/or *trh*, the expression of virulence associated traits including hemolysin, protease, motility, biofilm formation, and cytotoxicity correlated with increased temperature from 28 to 37°C. In contrast, the environmental isolates did not induce their virulence associated traits in response to a temperature of 37°C.

The occurence of *tdh* and/or *trh* genes among environmental *V. parahaemolyticus* isolates is typically 1–10%, but this depend on location, sample source and detection method. For example, we detected *tdh* and *trh* genes in 20.7 and 41.4% of the seafood samples, respectively, from southwest coast of India by PCR after 18 h enrichment in ST broth. In the same study, we isolated *tdh* and *trh-*carrying *V. parahaemolyticus* isolates from 19 to 44.8% of seafood samples, respectively, by colony hybridization following enrichment using ST broth (Raghunath et al., 2009). Kaysner et al. (1990) reported that between 49 and 78% of the sediment, water or oyster samples from Willapa Bay (WA, USA) contained *trh*-bearing *V. parahaemolyticus*. Alam et al. (2002) reported that *tdh* and *trh* genes were positive in 55 and 20% of environmental (water and sediment) samples, respectively, by MPN-PCR technique. But no *tdh* and/or *trh*-carrying strains were isolated by the conventional MPN-culture procedure. The *tdh* and *trh* genes are also present in non- *V. parahaemolyticus* vibrionaceae species such as *V. mimicus*, *V. cholerae* non-O1/non-O139, *V. hollisae*, *V. diaboilcus*, *V. alginolyticus,* and non-*vibrio* species such as *Aeromonas veronii* (Nishibuchi and Kaper, 1995; Gonzalez-Escalona et al., 2006; Raghunath et al., 2010; Shinoda, 2011; Klein et al., 2014). Such high frequency of these hemolysin genes in environmental strains of *V. parahaemolyticus* and occurance of these genes in the environmental strains of other *Vibrio* species indicate other potential roles of these hemolysins in the environment. Aquatic environment such as estuaries contain limited amount of nutrients. These hemolysins might be used to acquire nutrients through damage to cells of estuarine organisms. Matz et al. (2011) performed co-culture experiment of *V. parahaemolyticus* with a nanoflagellate *Cafeteria roenbergensis* and reported that the *tdh* gene is required for the persistence of *V. parahaemolyticus*.

## CONCLUSION

In conclusion, the reason for the selection of *V. parahaemolyticus* strains carrying *tdh* and/or *trh* in the human host and the role of these hemolysins in coordinating virulence associated gene expression in response to the environmental cues to facilitate colonization and infection needs to addressed. The reason for the high frequency of *tdh* and *trh* genes in environmental strains of *V. parahaemolyticus* is not clear. When compared to *tdh* gene, the detection rate of *trh* gene in clinical strains is very less but relatively more in environmental strains. Pathogenic potential of these environmental strains of *V. parahaemolyticus* carrying *trh* gene needs to be studied. Future studies should also focus on the role of TDH and TRH in the environmental strains of *V. parahaemolyticus* for contribution of environmental fitness.

## Conflict of Interest Statement

The author declares that the research was conducted in the absence of any commercial or financial relationships that could be construed as a potential conflict of interest.
